# Supplementary material for: Observation of hybrid higher-order skin-topological effect in non-Hermitian topolectrical circuits
Source: Nat Commun. 2021 Dec 10;12:7201. doi: 10.1038/s41467-021-26414-5 (PMC8664810; doi:10.1038/s41467-021-26414-5)
Supplement: Supplementary file 1 — Supplementary File [file 41467_2021_26414_MOESM1_ESM.pdf]

Observation of hybrid higher-order skin-topological effect in non-Hermitian topolectrical circuits

Deyuan Zou [1*], Tian Chen[1*+], Wenjing He[2], Jiacheng Bao[2], Ching Hua Lee[3], Houjun Sun[2+], and Xiangdong Zhang[1+]

[1]Key Laboratory of advanced optoelectronic quantum architecture and measurements of Ministry of Education, Beijing Key Laboratory of Nanophotonics & Ultrafine Optoelectronic Systems, School of Physics, Beijing Institute of Technology, 100081, Beijing, China

[2] Beijing Key Laboratory of Millimeter wave and Terahertz Techniques, School of Information and Electronics, Beijing Institute of Technology, Beijing 100081, China

[3] Department of Physics, National University of Singapore, Singapore 117542

*These authors contributed equally to this work. [+]Author to whom any correspondence should be addressed. E-mail: zhangxd@bit.edu.cn; sunhoujun@bit.edu.cn; chentian@bit.edu.cn

Supplementary Note 1. Measured results with different excitations

In our discussions of the main text, we chose to excite the site on the line 11 for convenience. Actually, other sites in the circuit can also be excited to obtain the results. We measure some results with different excited cites. The results are shown in Supplementary Figure 1.

Supplementary Figure 1(a) and 1(b) show the ST and SS effect with the excited site on the line 6. Supplementary Figure 1(c) and 1(d) show the ST and SS effect with the excited site on the line 1. We can see that voltage distributions at the resonance frequency for the hybrid second-order ST effect and SS effect are similar to the results shown in Supplementary Figure 3(c) and 3(d).

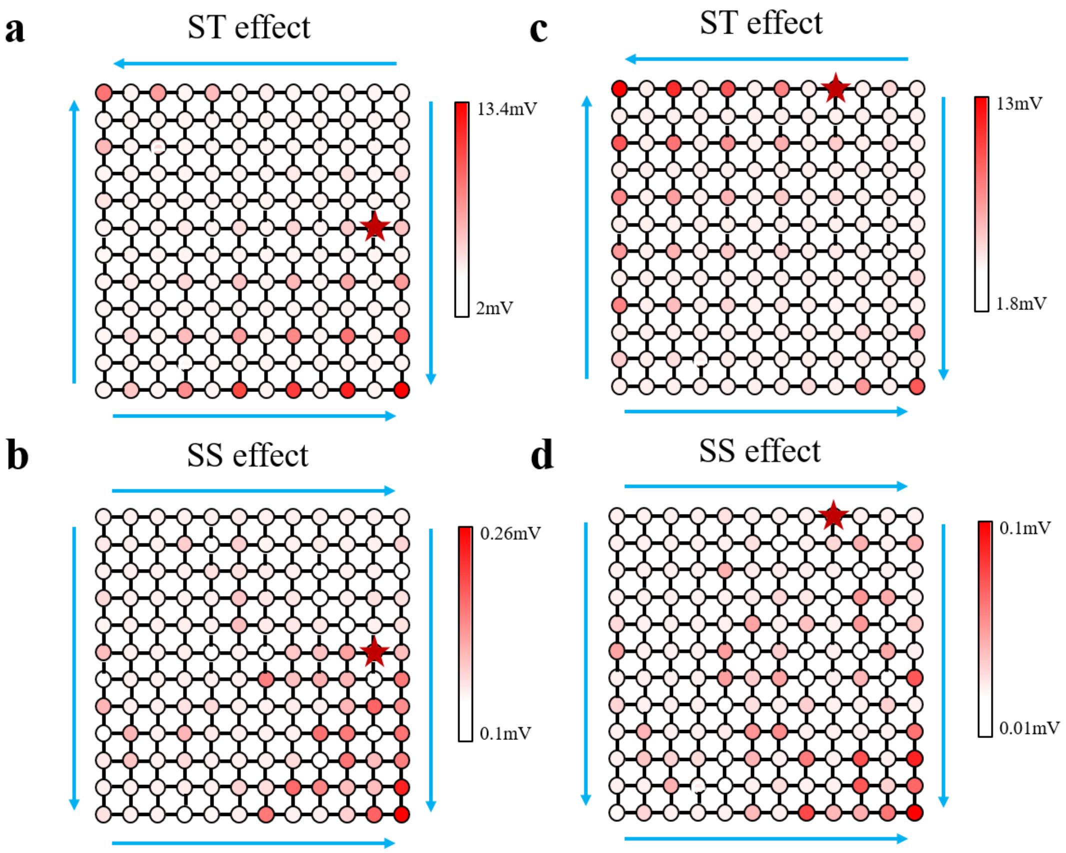

Supplementary Figure 1. Measured results with different excitations. Red stars are the points of voltage excitation. (a)-(b) The voltage distributions at the resonance frequency for the skin-topological (ST) and skin-skin (SS) effects with the excited site on the line 6. (c)-(d) The voltage distributions at the resonance frequency for the ST and SS effects with the excited site on the line 1.

Supplementary Note 2. The detailed comparisons for theoretical and experimental results

In this part, we provided detailed comparisons for theoretical and experimental results in both 2D and 3D circuits. The 2D results are shown in Supplementary Figure 2. We can see that in Supplementary Figure 2(a), voltages along right and left edges in both theoretical and experimental results are topological SSH localization. By contrast, the voltages in Supplementary Figure 2(b) are skin localization. Besides, theoretical and experimental results in ST and SS effect toward the same trend.

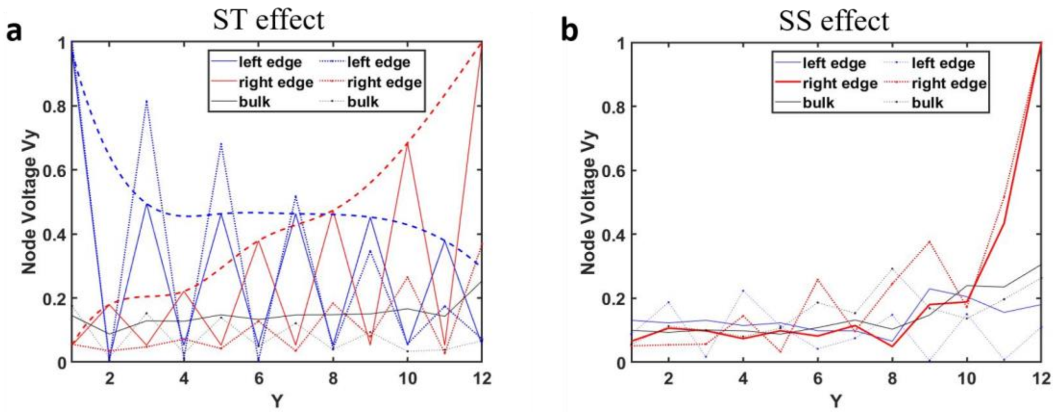

Supplementary Figure 2. The direct comparison for 2D theoretical and experimental results. Solid lines represent experimental results and dotted line represent theoretical results. (a) Comparison for skin-topological (ST) theoretical and experimental results. (b) Comparison for skin-skin (SS) theoretical and experimental results. Blue, red and black solid lines represent experimental voltage change along left edge, right edge, and average of bulk voltages in Fig. 3(c) and (d), respectively. Blue, red and black dotted lines represent theoretical voltage change along left edge, right edge, and average of bulk voltages in Fig. 2(c) and (d), respectively.

The 3D results are shown in Supplementary Figure 3. We can see that in Supplementary Figure 3(a), voltages in both theoretical and experimental results are topological SSH localization. By contrast, the voltages in Supplementary Figure 3(b) are skin localization. Besides, theoretical and experimental results in STT and SSS effect toward the same trend. The results mean that the experimental phenomena correspond exactly to the theoretical results, which indicates that the modes in 2D and 3D have been observed successfully in designed circuit systems.

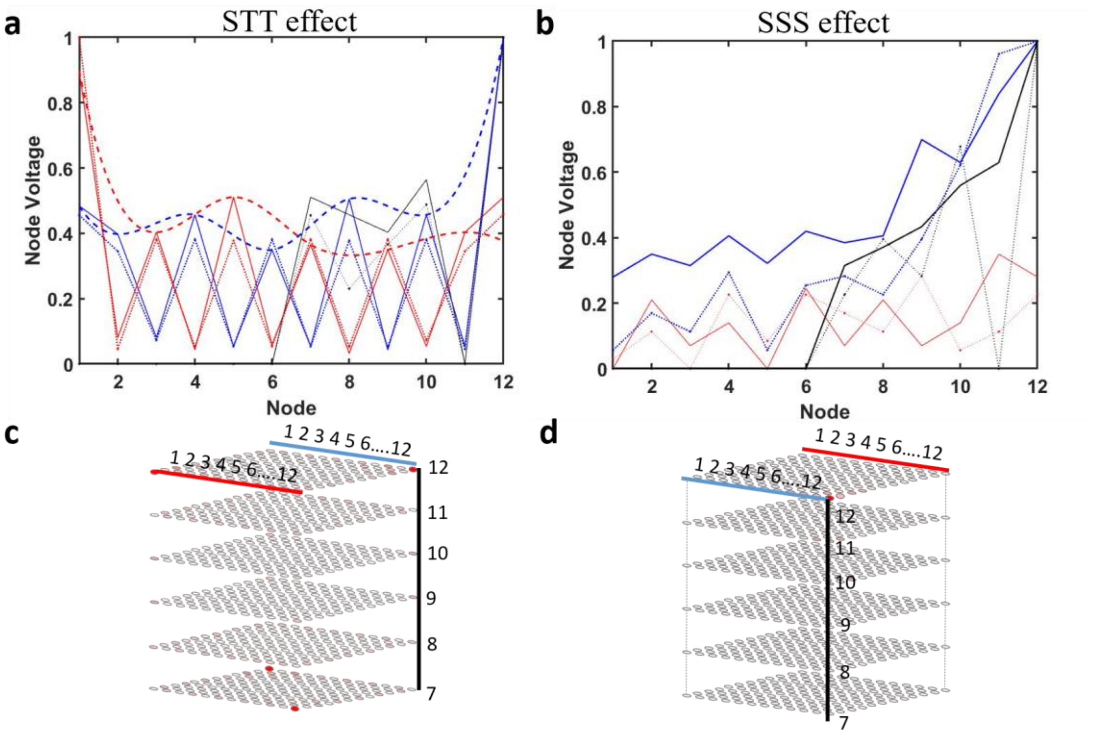

Supplementary Figure 3. The directly comparison for 3D theoretical and experimental results. Solid lines represent experimental results and dotted line represent theoretical results. (a) Comparison for skin-topological-topological (STT) theoretical and experimental results. (b) Comparison for skin-skin-skin (SSS) theoretical and experimental results. (c)-(d) show the corresponding colors for solid lines in (a) and (b), respectively.

Supplementary Note 3. Sample fabrications in the z direction

In this part, we show the details of the fabricated z-direction components for the 3D sample. The photographs are shown in Supplementary Figure 4. The right photograph shows the total INICs between two layers. The inset presents z-direction INICs in a unit cell.

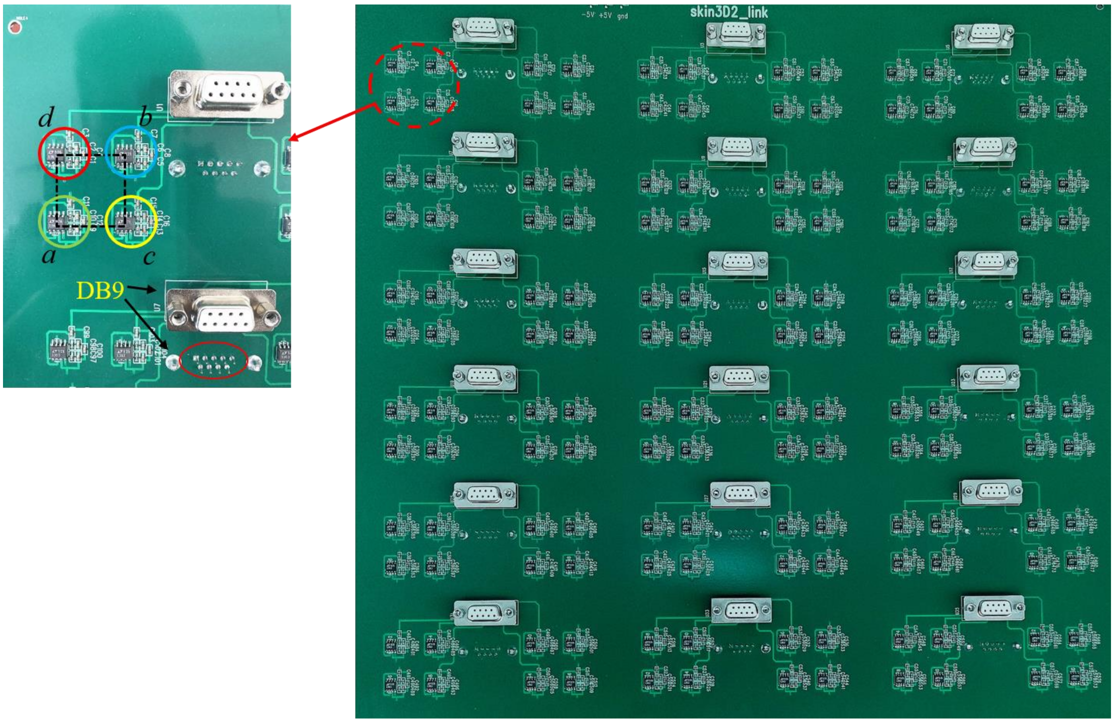

Supplementary Figure 4. The photograph of the fabricated z-direction components for the 3D sample. The inset presents the enlarged view. Each unit cell contains four sublattices (*a*, *b*, *c*, *d*). Four circles with different colors show z-direction INICs which connect corresponding points a, b, c and d between two x-y layers. We use DB9 to connect z-direction INIC with the upper and lower layers.

Supplementary Note 4. Figures for the method

A. Figure for 2D lattice model

In order to present the correspondence between the electric design in the main text and the lattice model, we provide the lattice Hamiltonian in Supplementary Figure 5. The green, blue, yellow and red spheres represent the sublattices *a*, *b*, *c* and *d* within unit cell plaquette. The details of unit cell are shown in Supplementary Figure 5.

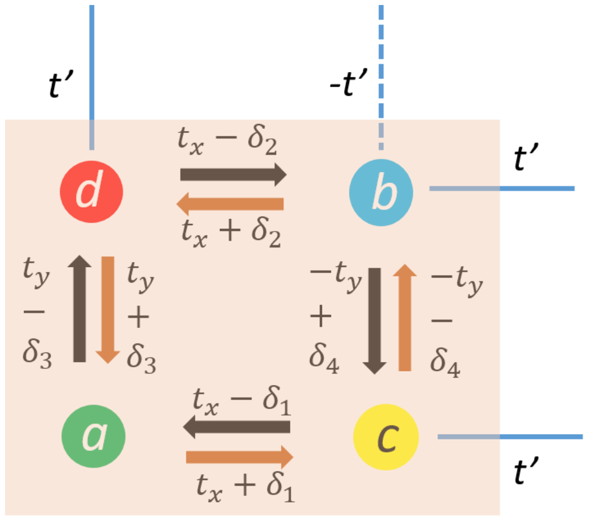

Supplementary Figure 5. The unit cell for the 2D lattice model. Each unit cell contains four sublattices (a, b, c, d). The brown and black lines with arrows represent asymmetric intra-cell coupling and the blue lines denote inter-cell coupling. This tight-binding model can demonstrate both hybrid second-order skin-topological (ST) effect and skin-skin (SS) effect with different values of $\delta_i (i = 1, 2, 3, 4)$.

B. Figure for 3D lattice model

These 3D electric circuits have the one-to-one correspondence to the lattices models. Here, we provide how to realize these 3D lattices by stacking 2D layers of lattices. The details of unit cell are shown in Supplementary Figure 6.

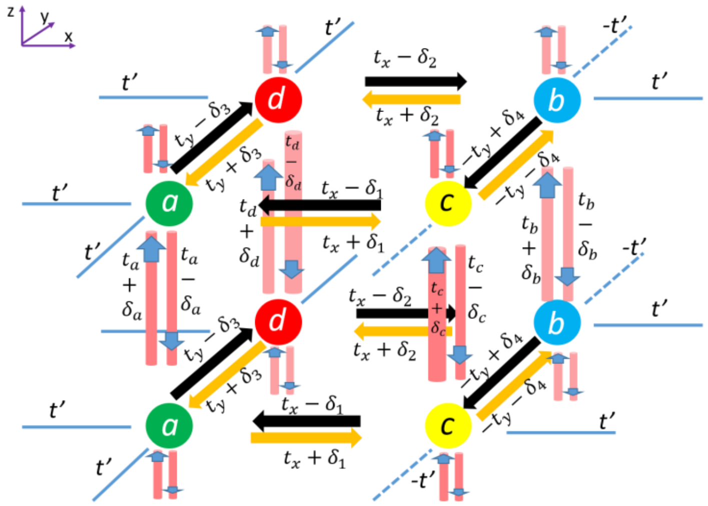

Supplementary Figure 6. The diagram for the 3D lattice model. We can see that each unit cell contains four sublattices (a, b, c, d). The brown and black lines with arrows represent asymmetric intra-cell coupling and the blue lines denote inter-cell coupling. The red lines with arrows represent asymmetric z

direction coupling. This tight-binding model can demonstrate both hybrid 3D skin-topological-topological (STT) effect and 3D skin-skin-skin (SSS) effect with different value of $\delta_i (i = 1, 2, 3, 4, a, b, c, d)$.

C. Figure for deriving circuit Laplacian in 2D electric circuit

Supplementary Figure 7 shows the model of unit cell for realizing the electric design to observe the SS mode in Fig. 1(c). There are voltages $V_a - V_d$ at the four nodes and currents $I_1 - I_8$ at the circuit branches.

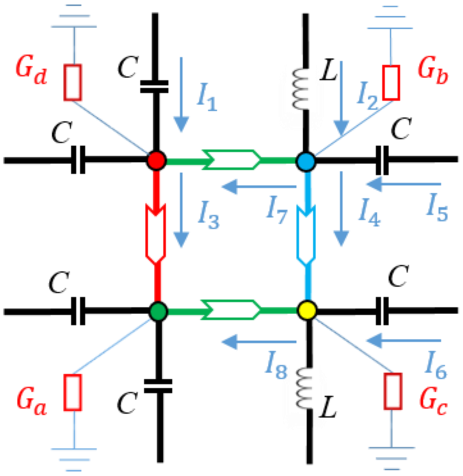

Supplementary Figure 7. The unit cell of proposed circuits to observe skin-skin (SS) mode. Each unit cell contains four sublattices (*a*, *b*, *c*, *d*). The arrows mark the direction of currents. Arrows with different colors are the same as INICs labeled in Fig. 1(d). $G_i (i = a, b, c, d)$ are the grounding parts.

Supplementary Note 5. Grounding of the finite electric circuit

To fulfill the lattice Hamiltonian by a topolectrical circuit Laplacian, we need to choose the suitable grounding to eliminate the diagonal elements of open circuit Laplacian at the resonant frequency. This can be easily realized by making the inductivities and capacitances enter the circuit Laplacian with opposite sign, that is,

$$J(\omega) = i\omega C - \frac{i}{\omega L} \tag{1}$$

Hence, the contribution of inductivities (capacitances) at the fix node can be cancelled by grounding matched capacitances (inductivities). We present the grounding patterns in Supplementary Table 1-4, which correspond to the actual sample.

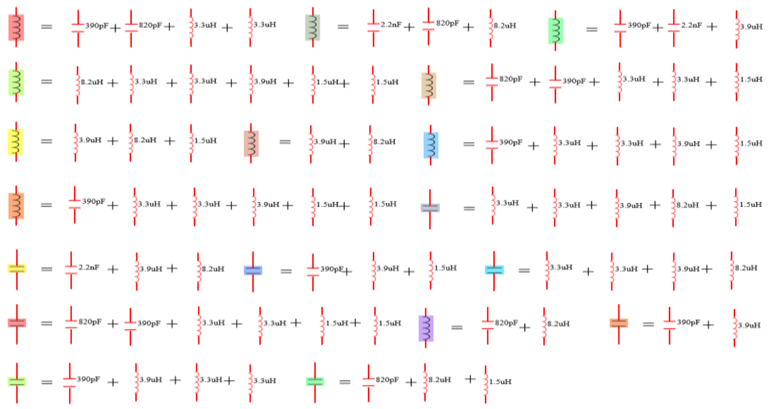

Supplementary Table 1

Supplementary Table 2

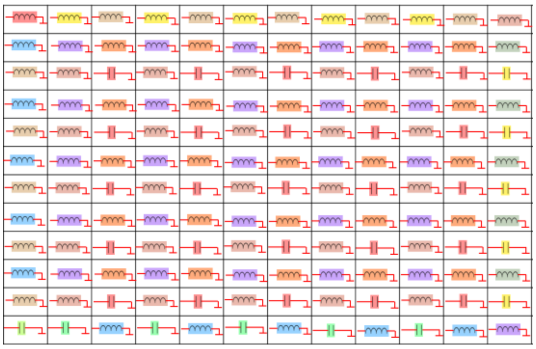

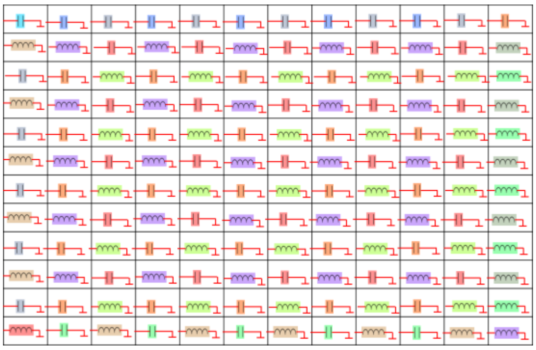

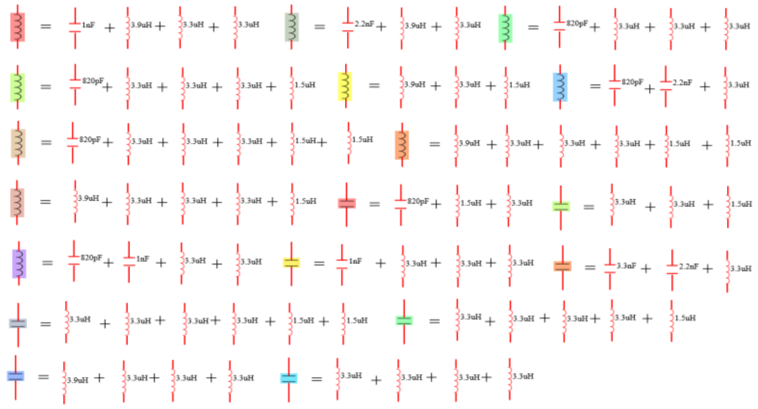

Supplementary Table 3

End

Mid

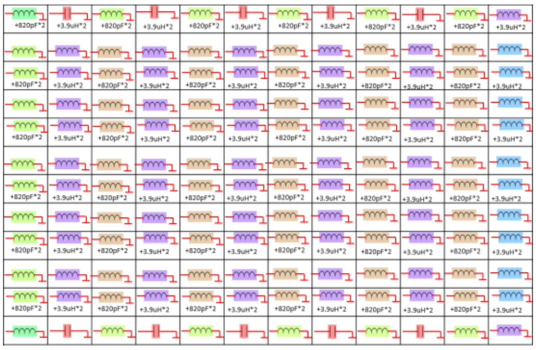

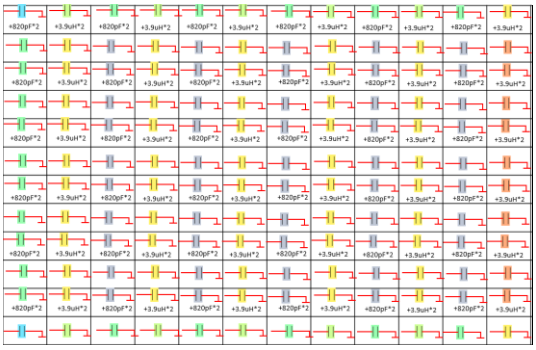

Top

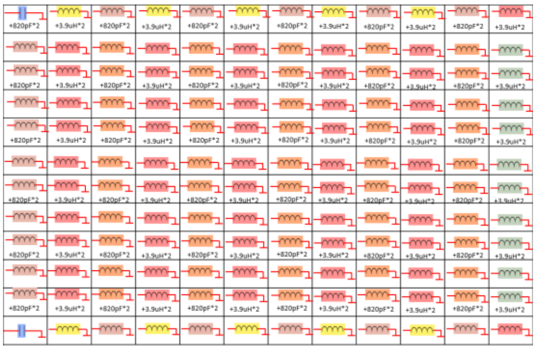

Supplementary Table 4

End

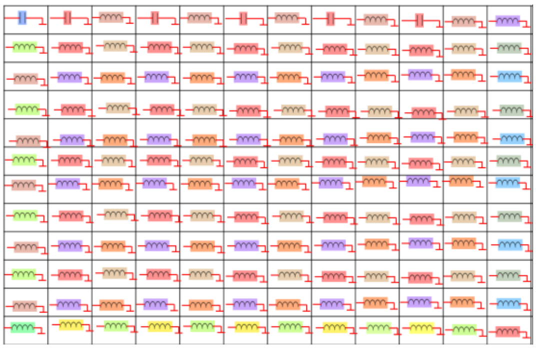

Mid

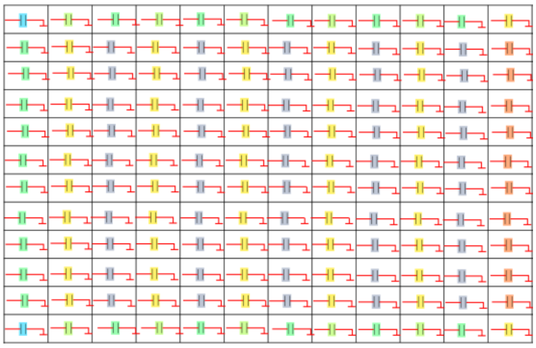

Top

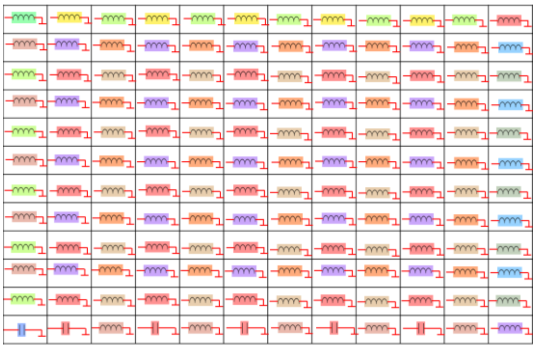

Supplementary Table 1 is grounding part for SS effect. Supplementary Table 2 is grounding part for hybrid second-order ST effect. Supplementary Table 3 is grounding part for 3D SSS effect. Supplementary Table 4 is grounding part for hybrid 3D STT effect. As we mentioned above, the whole 3D sample is divided to eleven layers. Only six layers have grounding parts. The end, mid and top are for the end layer, mid 2-4 layers and top layer, respectively.
